# Gelatin/Ascorbic Acid Scaffolds for Controlled Release of Allantoin: A Fully Natural Approach for Skin Tissue Regeneration Through Pro-Regenerative, Antimicrobial, and Keratinocyte-Supportive Properties

**DOI:** 10.3390/pharmaceutics18030391

**Published:** 2026-03-22

**Authors:** Marija M. Babić Radić, Marija Vukomanović, Martina Žabčić, Lea Gazvoda, Dubravka Živanović, Simonida Tomić

**Affiliations:** 1 University of Belgrade, Innovation Center of Faculty of Technology and Metallurgy, Karnegijeva 4, 11000 Belgrade, Serbia; 2 Advanced Materials Department, Jožef Stefan Institute, Jamova 39, 1000 Ljubljana, Slovenia; marija.vukomanovic@ijs.si (M.V.); martina.zabcic@ijs.si (M.Ž.); lea.gazvoda@ijs.si (L.G.); 3 Jozef Stefan International Postgraduate School, 1000 Ljubljana, Slovenia; 4 University of Belgrade, University Clinical Center of Serbia, Clinic of Dermatology and Venereology, Pasterova 2, 11000 Belgrade, Serbia; dubravka.zivanovic@med.bg.ac.rs; 5 University of Belgrade, Faculty of Technology and Metallurgy, Karnegijeva 4, 11000 Belgrade, Serbia; simonida@tmf.bg.ac.rs

**Keywords:** gelatin/ascorbic acid-based scaffolds, crosslinking gelatin with ascorbic acid, scaffolds enriched with allantoin, scaffold-mediated skin tissue regeneration, controlled release of allantoin

## Abstract

**Background/Objectives**: Nature-inspired therapeutic strategies that promote biological regenerative mechanisms and replicate the native structural microenvironment conductive to formation of healthy tissue are increasingly recognized as a promising platform for skin tissue regeneration and wound healing. This study proposes an innovative design of novel multifunctional scaffolds composed entirely of natural components—gelatin, L-ascorbic (ASA) acid and allantoin—as a bioinspired approach for skin tissue regeneration through pro-regenerative, antimicrobial, and keratinocyte-supportive properties. **Methods**: The biocompatible, skin-adhesive scaffolds were prepared via a simple and environmentally friendly heat-induced crosslinking of gelatin with varying ASA contents, and by enriching the system with allantoin. The influence of ASA content on scaffold properties was investigated through characterization of their morphology, porosity, swelling behavior, skin tissue adhesion, and allantoin release potential. Biocompatibility was evaluated in vitro using human keratinocyte (HaCaT) cells, while antibacterial activity was assessed against *Escherichia coli* and *Staphylococcus epidermidis*. **Results**: The scaffolds revealed a highly porous, interconnected structure with tunable porosity (87.37–92.39%) and soft-tissue-matched mechanical properties (0.81–1.47 MPa). Incorporation of allantoin into the scaffolds enhanced their mechanical performance and swelling capacity. All scaffolds demonstrated antibacterial activity against both tested bacteria, supported keratinocyte viability and provided sustained release of allantoin for up to 76 h, confirming their multifunctional pro-regenerative potential. **Conclusions**: The novel gelatin/ascorbic acid scaffolds enriched with allantoin combine a porous replicated structure of native extracellular matrix, fluid absorption capacity, soft-tissue-like mechanical properties, stable skin tissue adhesion, cytocompatibility and antibacterial functionality with the pro-regenerative properties of allantoin, thereby representing a multifunctional and biologically inspired platform for advanced skin tissue regeneration and wound-healing applications.

## 1. Introduction

Skin tissue regeneration and wound healing are highly dynamic biophysiological processes involving a cascade of cellular and molecular events that ultimately require a multifaceted therapeutic approach [1,2]. Conventional strategies for skin tissue regeneration and wound management have primarily focused on the controlled and targeted release of bioactive agents, aiming to reduce infection and stimulate tissue repair through pharmacological activity [3]. However, such approaches often fail to address the critical need for three-dimensional (3D) structural support during tissue regeneration, which is essential for guiding cellular processes and enabling effective integration, growth and reconstruction of functional tissue through the replicated structure of the extracellular matrix (ECM) [4]. In this context, tissue-engineering strategies based on scaffolding biomaterials have emerged as a promising approach to overcome these limitations by combining the controlled release of bioactive agents with a biomimetic 3D architecture that supports adhesion, proliferation, and migration of cells, and ECM synthesis directly at the site of tissue defect [4,5]. The efficacy of these therapeutic scaffold-based systems strongly depends on the physicochemical and biological properties of the scaffolding biomaterials, including their biocompatibility, porosity, mechanical and adhesive characteristics and capacity for incorporation and release of bioactive agents, thereby providing simultaneous structural and biological support for tissue repair.

Natural polymer-based scaffolds are of particular interest in the field of tissue engineering due to their inherent biocompatibility, biodegradability, and ability to replicate key features of the native ECM [6,7,8,9,10]. These scaffolds are capable of providing a porous, hydrated, and mechanically compliant microenvironment that promotes adhesion, migration and proliferation of cells, thereby facilitating tissue regeneration at the wound site [6]. One of the promising natural polymers for tissue regeneration applications is gelatin, a denatured form of collagen, due to its biochemical similarity to the native ECM and its favorable biological profile, including the presence of Arg-Gly-Asp (RGD) sequences that support cellular functions and processes [4,11]. Gelatin-based biomaterials are biodegradable through enzymatic proteolysis, highly biocompatible and have no antigenicity under physiological conditions, which makes them suitable for supporting cellular processes and matrix remodeling [12,13]. Moreover, numerous studies have confirmed the potential of gelatin-based biomaterials to provide a microenvironment favorable for various types of cells, including fibroblasts, endothelial cells, and cardiac cells, thereby promoting repair and regeneration of damaged tissue [13,14]. In skin tissue regeneration and wound healing, gelatin-based scaffolds have been explored for their ability to maintain a moist environment, absorb exudate, and act as temporary ECM substitutes that guide 3D skin tissue reconstruction [4]. Nevertheless, gelatin exhibits limited mechanical stability and high sensitivity to aqueous environments, which often requires the implementation of effective and biocompatible design strategies to ensure structural integrity under physiological conditions. Due to its hydrophilic nature and the presence of numerous polar functional groups, gelatin can absorb large amounts of water or physiological fluids, leading to a high degree of swelling and a reduction in mechanical strength in hydrated conditions. This extensive fluid uptake weakens intermolecular interactions within the polymer network and may compromise the structural integrity of gelatin-based materials. Because of these limitations, numerous studies have focused on improving the mechanical properties of gelatin-based materials through crosslinking strategies or composite design. Conventional crosslinking agents such as glutaraldehyde and carbodiimides have been shown to significantly increase tensile strength and compressive modulus while simultaneously reducing swelling capacity [12]. Similarly, combining gelatin with other polymers, such as polysaccharides or synthetic polymers, or with inorganic compounds that act as fillers such as nanoparticles of graphene oxide (GO), titanium dioxide (TiO_2_), or zinc oxide (ZnO), it is possible to provide stronger mechanical properties and adequate flexibility that allow easy handling during wound-healing treatment or tissue-engineering applications [9,15,16,17]. Thus, a critical aspect in the development of functional gelatin-based scaffolds lies in the careful design and selection of the crosslinking process. While conventional chemical crosslinkers such as glutaraldehyde or carbodiimides can improve gelatin stability, the potential toxicity associated with their residuals raises concerns due to compromised biocompatibility and clinical safety [18,19,20]. This has prompted increasing efforts toward alternative, safer crosslinking strategies, including the use of enzymatic, physical, or natural crosslinkers, aiming to preserve the favorable biological profile of gelatin while minimizing safety risk. In this regard, L-ascorbic acid (ASA), which is an antioxidant and an essential cofactor in collagen biosynthesis, has great potential to be used as a natural, safe, and biocompatible crosslinker for gelatin, with additional benefits for ECM remodeling and skin tissue regeneration [21,22,23]. Crosslinking of gelatin with ASA occurs through Maillard reactions involving amino groups of gelatin, primarily those of lysine, arginine, and histidine. Initially, ASA reacts with the ε-amino group of gelatin to form 3-deoxy-3-(alkylamino)ascorbic derivatives, which subsequently oxidize into L-dehydroascorbic acid (DHA) [24,25]. This primary oxidation product of ASA (DHA) can act as a natural crosslinking agent for gelatin due to the presence of two reactive carbonyl groups on its molecule, which may form covalent bonds with amine groups from gelatin [26]. Unlike conventional crosslinkers, ASA and DHA released from the scaffold are not expected to cause cytotoxic effects, as their biocompatibility is well established [27,28]. Additionally, DHA possesses the potential to promote collagen deposition in formation of connective tissue and also possesses antitumor activity, partly through kinase inhibition and intracellular reduction to ascorbic acid [28,29].

To further enhance scaffold performance for skin tissue regeneration and wound healing, the incorporation of bioactive agents into the scaffold system represents a promising strategy. Allantoin is a naturally occurring bioactive compound with well-documented beneficial effects on the wound-healing and skin regeneration processes [30]. Numerous studies have demonstrated its ability to stimulate local granulation, accelerate the healing of chronic wounds, modulate inflammatory responses, promote fibroblast proliferation, and enhance ECM synthesis, thereby supporting more organized skin tissue regeneration [30,31,32]. Among great biocompatibility and pro-regenerative activity, allantoin has also demonstrated antibacterial activity, which makes it favorable for the management of infected wounds or skin injuries [33]. The combination of a multifunctional scaffold with the pro-regenerative properties of allantoin presents a promising strategy for developing advanced polymeric systems for skin tissue regeneration and wound management.

In this study, we hypothesize that a scaffold composed exclusively of natural components—gelatin, L-ascorbic acid, and allantoin—can serve as a multifunctional biomaterial for skin tissue regeneration. The porous gelatin-based matrix is expected to provide structural support for skin tissue regeneration by mimicking the architecture of the natural extracellular matrix (ECM) directly at the wound site, while the incorporation of allantoin may promote regenerative processes through its localized release. In addition, crosslinking gelatin with L-ascorbic acid is expected to improve scaffold stability while maintaining biocompatibility and reducing the potential toxicity associated with conventional chemical crosslinkers. Together, these features may enable the scaffold to simultaneously provide structural support at the wound site and bioactive stimulation of skin tissue regeneration. Gelatin-based scaffolds were fabricated via a simple and environmentally friendly reaction based on heat-induced crosslinking of gelatin with varying contents of ASA, and by enriching the system with allantoin. Various amounts of ASA were used to evaluate their influence on gelatin-based scaffold properties, including morphology, porosity, swelling behavior, adhesiveness to skin tissue, and allantoin release potential. Biocompatibility of the scaffolds and their ability to support cell proliferation were evaluated in vitro on a human keratinocyte cell line (HaCaT). Antimicrobial activity was assessed against both Gram-positive *Staphylococcus epidermis* (*S. epidermidis*) and Gram-negative bacteria *Escherichia coli* (*E. coli*). Furthermore, in vitro allantoin release studies confirmed the potential of the scaffolds for sustained release of bioactive agents. The proposed scaffolds are designed to provide 3D structural support through replicated ECM structure directly at the defect tissue site, promote intrinsic regenerative processes, including keratinocyte proliferation, and enable sustained release of allantoin, thus representing a multifunctional and biologically inspired platform for advanced skin tissue regeneration and wound-healing applications.

## 2. Materials and Methods

### 2.1. Materials

Gelatin from porcine skin (G, Type A, bioreagent), L-ascorbic acid (ASA, 99%), and allantoin (All, ≥98%) were all purchased from Sigma-Aldrich (St. Louis, MO, USA). Dulbecco’s modified Eagle’s medium (DMEM, GibcoTM, Thermo Fisher Scientific, Waltham, MA, USA), fetal bovine serum for cell culture (FBS, GibcoTM, Thermo Fisher Scientific, Waltham, MA, USA), penicillin–streptomycin antibiotics (Gibco, Grand Island, NY, USA), Dulbecco’s phosphate-buffered saline (DPBS, GibcoTM, Thermo Fisher Scientific, Waltham, MA, USA), and Presto Blue Cell Viability Reagent (Molecular Probes, Invitrogen, Thermo-Fisher Scientific, Waltham, MA, USA) were used for cytotoxicity assessment. For buffer preparation, potassium hydrogen phosphates (Sigma-Aldrich, ST. Louis, MO, USA) were used, while all synthesis reactions were performed using lab-produced, ultra-distilled water.

### 2.2. Scaffolds Preparation

Two series of scaffolds (S)—SG/ASA (the scaffolds based on gelatin and ascorbic acid) and SG/ASA/All (the scaffolds based on gelatin and ascorbic acid and enriched with allantoin)—were developed for potential applications in skin tissue regeneration and wound healing. L-ascorbic acid was used as a green, biocompatible, and eco-friendly crosslinker for gelatin, offering a sustainable alternative to conventional chemical crosslinkers such as glutaraldehyde or carbodiimide, which may compromise the biocompatibility and overall safety of the final biomaterial [18,19,20]. For the scaffold synthesis, gelatin and varying amounts of ASA (Table 1) were dissolved in 40 mL of ultra-distilled water, thoroughly stirred, and heated at 70 °C for 5 h to promote crosslinking reactions. The content of ASA (Table 1) was varied to evaluate its influence on scaffold properties, including morphology, porosity, swelling behavior, adhesiveness to the skin tissue, biocompatibility and the potential for controlled release of allantoin. In order to enhance the bioactivity of the scaffolds, allantoin was selected as an active agent suitable for skin tissue regeneration and was incorporated into the scaffolds. The resulting homogeneous reaction mixtures were poured into Petri dishes and stored at 4 °C for 24 h to complete the crosslinking process. Afterward, the samples were washed with ultra-distilled water to remove unreacted residues, allowed to swell, frozen at −80 °C, and finally lyophilized to obtain porous and stable scaffolds.

### 2.3. Hydrogel Scaffold Characterization

#### 2.3.1. Fourier-Transform Infrared Spectroscopy (FTIR)

The chemical composition of the scaffolds was evaluated through FTIR spectroscopy, with FTIR spectra collected over a wavelength range of 700–4000 cm^−1^, using a Thermo-Scientific Nicolet 6700 FTIR spectrometer (Thermo Fisher Scientific, Uppsala, Sweden) equipped with a diamond crystal and the ATR sampling method.

#### 2.3.2. Scanning Electron Microscopy (SEM)

A scanning electron microscope (SEM, Thermo Fisher Quanta 650 ESEM, Tokyo, Japan) was used to analyze the surfaces and cross-sectional morphologies of the scaffolds. Before SEM imaging, the lyophilized scaffolds were coated with a layer of gold using a BAL-TEC SCD 005 sputter-coater (Balzers, Liechtenstein).

#### 2.3.3. Porosity Measurements

Porosity was determined by measuring the real volume and density using a ULTRApyc 5000 pycnometer (ANTON PAAR, Graz, Austria) with a small insert chamber calibrated to a nanosphere weighing 0.0898 g. Each individual sample underwent five measurements, and the porosity was calculated based on the average of the three most similar values. The measurement was performed at a target pressure of 3 psi using helium as the displacement gas. Each value for the sample was performed on three parallels.

#### 2.3.4. Mechanical Testing

Dynamic mechanical analysis (DMA) was performed on Mettler Toledo DMA/SDTA861e to determine the compression modulus (E) of prepared scaffolds in the elastic region. The modulus was confirmed by verifying the linear relationship between stress and strain and ensuring a consistent measured value across different displacements and forces. Measurements were conducted within a force range of 0.5 N to 1.5 N, under a constant deformation pressure of 2 N, at a frequency of 2 Hz, with data recorded at 0.05 N increments. The compression modulus for one of three parallel samples was calculated as the average of the measured values over this range. For each condition, three samples were tested to determine the average value and standard deviation within the same batch.

### 2.4. In Vitro Swelling Study

The capacity of the scaffolds to absorb biological fluids was evaluated in a phosphate-buffered solution (pH 7.4) maintained at 37 °C by the procedure previously described in [34,35,36,37,38].

### 2.5. Adhesiveness Test

The ability of the prepared scaffolds to adhere to skin tissue was assessed by fixing swollen samples onto skin tissue at a mobile joint and monitoring their stability during joint motion, with flexion angles ranging from 0° to 120° [38]. The scaffold attachment was photographed at various joint positions, including complete extension (0°) and progressive bending at 45°, 90°, and 120°, as well as during adhesion to the palmar surface of a finger.

### 2.6. Biocompatibility Probes

#### 2.6.1. In Vitro Cytotoxicity Assessment

Human keratinocyte (HaCaT) cells (TCC PCS-200-011) were cultivated in full growth medium (containing Dulbecco’s modified Eagle’s medium, 10% fetal bovine serum for cell culture, and 1% penicillin–streptomycin antibiotics) and seeded in 24-well plates (NunclonTM, Thermo Scientific, Waltham, MA, USA). The cells were incubated at 37 °C and 5% CO_2_ (MCO-19AIC(UV)-PE incubator, Panasonic, Osaka, Japan) to confluency. The samples were first ground in nitrogen to obtain a powder. Then, 3 mg of the samples was dispersed/dissolved in 900 µL of full growth media in an ultrasonic water bath for 20 min (Elma). Then, 300 µL of the suspension/solution was added per well (1 mg per well) to confluently grow cells and incubated overnight (exposure for 20–24 h). The next day, the cells were washed with Dulbecco’s phosphate-buffered saline, and a fresh medium with 10% Presto Blue Cell Viability Reagent was added to the cells. The cell viability was assessed by fluorescence measurement at 560/590 nm Ex/Em (Synergy H1 plate reader, Biotek, Winooski, VT, USA) after 1 h of incubation. All samples were performed in three separate wells for statistical analysis.

#### 2.6.2. Antimicrobial Activity

The scaffolds were first compressed between two metal disks under a 50 kN force to obtain flattened disks. These disks were then cut into four pieces and weighed, with each sample weighing approximately 20–40 mg. On pre-prepared LB agar plates, 50 µL of E. coli or S. epidermidis bacterial suspension, adjusted to a concentration of 10^8^ CFU/mL, was evenly spread. The scaffold pieces were then placed on top and incubated for 20–24 h. After incubation, the plates were collected and imaged using the Acolyte automated colony-counting machine.

#### 2.6.3. In Vitro Simultaneous Controlled Release Study

The scaffolds’ potential to release allantoin was evaluated using a UV–vis spectrophotometer (Shimadzu UV-1800, Shimadzu Corporation, Kyoto, Japan). The scaffolds enriched with allantoin SG/ASA/All were immersed in a basket stirrer containing 800 mL of phosphate buffer (pH 7.4) maintained at 33.5 °C to mimic physiological conditions. The amount of allantoin released over time was determined by measuring the absorbance of the release medium at predetermined intervals, with the maximum absorption wavelength (λmax) set at 204 nm [39].

### 2.7. Statistical Analysis

Experimental data are presented as means  ±  SD based on three independent replicates. Statistical analyses were performed using GraphPad Prism version 9.0.0 (121). Multiple-comparison one-way ANOVA was used to assess the significance of differences between groups. A *p*-value of less than 0.05 (*p*  <  0.05) was considered statistically significant.

## 3. Results and Discussion

### 3.1. Preparation of the Scaffolds

**Scheme 1:**
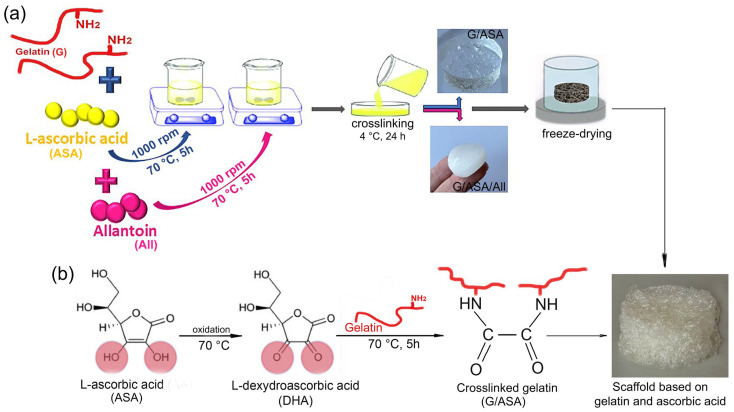
Summary of gelatin-based scaffold preparation. (**a**) Schematic representation of synthesis route of the scaffold with or without allantoin. (**b**) Potential pathway of the crosslinking reaction between gelatin and L-ascorbic acid.

Inspired by the essential role of ECM in maintaining the structural and mechanical integrity of skin tissue, as well as by the need for bioactive support during skin tissue regeneration, this study presents the design of a novel biomaterial for skin tissue regeneration composed exclusively of natural components: gelatin, L-ascorbic acid, and allantoin. In order to replicate the natural ECM, gelatin was selected as the base material for constructing a 3D porous scaffold due to its biocompatibility and structural similarity to collagen, while ASA was used as a natural crosslinking agent to minimize the potential toxicity associated with residues from conventional crosslinkers [18,19,20]. To enhance the bioactivity of the scaffolds, a natural bioactive compound—allantoin—was used for incorporation into the scaffolds due to its excellent biocompatibility and well-documented ability to promote wound healing and skin tissue regeneration by stimulating fibroblast proliferation, modulating the inflammatory response, enhancing ECM synthesis, and providing additional antibacterial activity beneficial for the treatment of chronic and infected wounds and skin injuries [30,31,32,33]. Gelatin-based scaffolds were fabricated via a simple and environmentally friendly approach through heat-induced crosslinking of gelatin by varying the contents of ASA and by enrichment with allantoin, as illustrated in Scheme 1a. The content of ASA was varied in order to evaluate its influence on the key scaffold properties, including morphology, porosity, swelling behavior, adhesion to the skin tissue, biocompatibility and allantoin release potential. A potential pathway of the crosslinking reaction between gelatin and ASA is presented in Scheme 1b. In the initial step of crosslinking, ASA is converted into reactive carbonyl compounds such as DHA, which can readily participate in Maillard-type reactions with the ε-amino groups of gelatin [40]. This primary oxidation product of ASA can act as a natural crosslinking agent for gelatin due to the presence of two reactive carbonyl groups on its molecule, which may form covalent bonds with amine groups from gelatin [26]. These carbonyl–amine reactions lead to covalent crosslinking between gelatin chains. Obtaining covalent crosslinking of gelatin enhances the structural stability of the gelatin-based scaffolds under physiological conditions and contributes to the overall mechanical reinforcement of the scaffolds.

### 3.2. Structural Characteristics of the Scaffolds—FTIR Analysis

**Figure 1:**
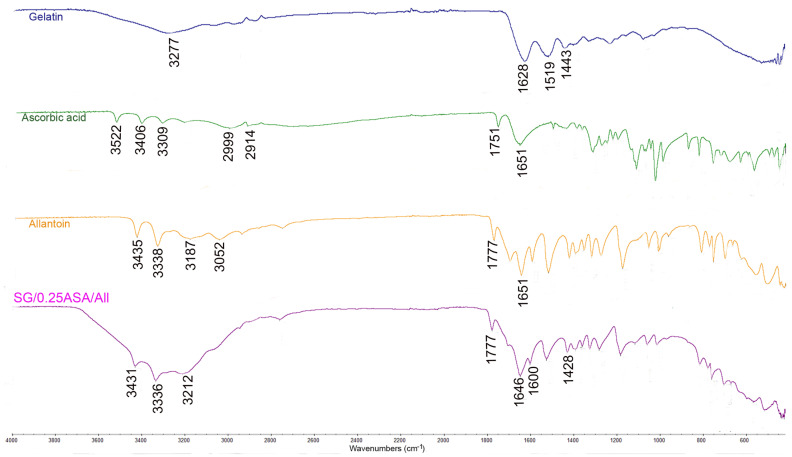
FTIR spectra of pure gelatin (G), L-ascorbic acid (ASA), and allantoin (All), and the scaffold based on gelatin and ascorbic acid enriched with allantoin (SG/0.25ASA/All).

FTIR spectroscopy was used to investigate the molecular interactions between gelatin and ASA and to confirm the incorporation of allantoin within the scaffold structure. The FTIR spectra of the pure scaffolds’ components, including gelatin, ASA, and allantoin, as well as of the synthesized gelatin/ascorbic acid scaffold enriched with allantoin (SG/0.25ASA/All), are presented in Figure 1. The FTIR spectrum of pure gelatin exhibited absorption bands at around 1628 cm^−1^, 1519 cm^−1^, and 1443 cm^−1^, which are attributed to the C=O stretching vibrations of peptide bonds (amide I), N–H bending vibrations coupled with C–N stretching (amide II), and C–N stretching combined with N–H bending vibrations (amide III), respectively [37]. Additionally, the broad absorption band centered around 3277 cm^−1^ is attributed to O–H stretching vibrations associated with hydroxyl groups present in the gelatin [16,41]. The FTIR spectrum of pure ASA shows bands associated with O–H stretching vibrations (around 3200–3500 cm^−1^), and C=O stretching (around 1751 cm^−1^) [42]. The FTIR spectrum of allantoin exhibited characteristic IR absorption bands, including N–H stretching vibrations at around 3435 cm^−1^ and 3338 cm^−1^ and carbonyl (C=O) stretching vibrations near 1777 cm^−1^ and 1651 cm^−1^, which are commonly attributed to the ureide functional groups of the molecule [43].

Compared with the FTIR spectrum of pure gelatin, the FTIR spectrum obtained for the synthesized scaffold SG/0.25ASA/All displayed changes in amide regions, particularly amide I and amide II, suggesting the occurrence of gelatin crosslinking. Evidence of crosslinking gelatin with ASA was observed through a slight shift of the gelatin characteristic absorption bands toward lower wavenumbers (from 1628 cm^−1^ to 1600 cm^−1^ for amide I and from 1519 cm^−1^ to 1428 cm^−1^ for amide II), accompanied by a reduced intensity of peaks, indicating the involvement of gelatin amino groups in molecular interactions and a decrease in the number of free amide functionalities of gelatin. In addition, a new peak was detected in the FTIR spectrum of SG/0.25ASA/All at around 1646 cm^−1^, which is attributed to the C=O stretching of carboxyl groups introduced through interactions with ASA. Furthermore, the disappearance of the C–H stretching absorption bands at 2999 cm^−1^ and 2914 cm^−1^, which are characteristic bands of ASA, indicates that ASA is not present in its free form in the scaffold but is probably chemically bound within the gelatin network. The successful incorporation of allantoin into the scaffold was confirmed by the appearance of a novel absorption band in the FTIR spectrum of SG/0.25ASA/All at approximately 1777 cm^−1^. This absorption band is attributed to the C=O stretching vibrations of the ureide functional group characteristic of allantoin [43,44]. The observed spectral shifts and the presence of characteristic allantoin absorption bands confirm both effective gelatin crosslinking and incorporation of the bioactive agent into the scaffold.

### 3.3. Morphology of the Scaffolds—SEM Analysis

**Figure 2:**
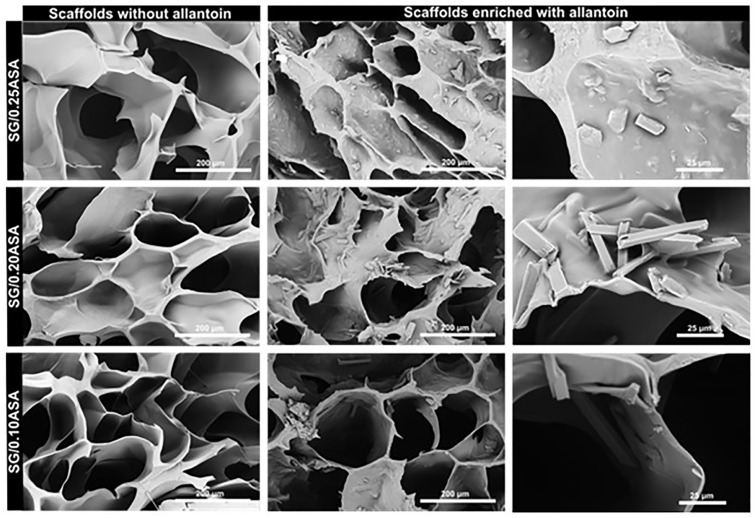
SEM micrographs of the cross-section of the scaffolds based on gelatin/ascorbic acid—SG/0.25ASA, SG/0.20ASA, and SG/0.10ASA—and scaffolds based on gelatin/ascorbic acid enriched with allantoin—SG/0.25ASA/All, SG/0.20ASA/All, and SG/0.10ASA/All.

The microstructural morphology of the gelatin/ascorbic acid scaffolds (SG/ASA) and those enriched with allantoin (SG/ASA/All) was examined by scanning electron microscopy (SEM), and the obtained micrographs are presented in Figure 2. The micrographs of all samples reveal a highly porous microstructure with uniformly shaped and interconnected pores, which is an essential parameter for efficient tissue regeneration as it supports cellular processes and ECM deposition, while also enabling efficient transport of nutrients, oxygen, and bioactive agents through the scaffold [45]. As can be seen from Figure 2, the scaffolds enriched with allantoin possess thicker pore walls with a rougher texture compared with the scaffolds without allantoin, indicating the successful incorporation of the bioactive compound. This phenomenon can be attributed to the fact that the incorporation of allantoin may alter the viscosity and phase separation behavior of the precursor solution during scaffold formation, thereby influencing pore architecture and wall morphology [46,47]. Such effects have been previously reported for other biomaterial systems, where the addition of bioactive molecules can modify polymer chain organization and scaffold microstructure [46,47]. In addition, small granules/elongated structures were observed within the pore walls of the scaffolds enriched with allantoin, further confirming the presence and homogeneous distribution of allantoin within the scaffold structure. The obtained porous morphology of the scaffolds is expected to promote favorable cell–material interactions and thereby enhance the overall regenerative potential of the scaffolds for skin tissue regeneration and wound-healing applications.

### 3.4. Porosity of the Scaffolds

**Figure 3:**
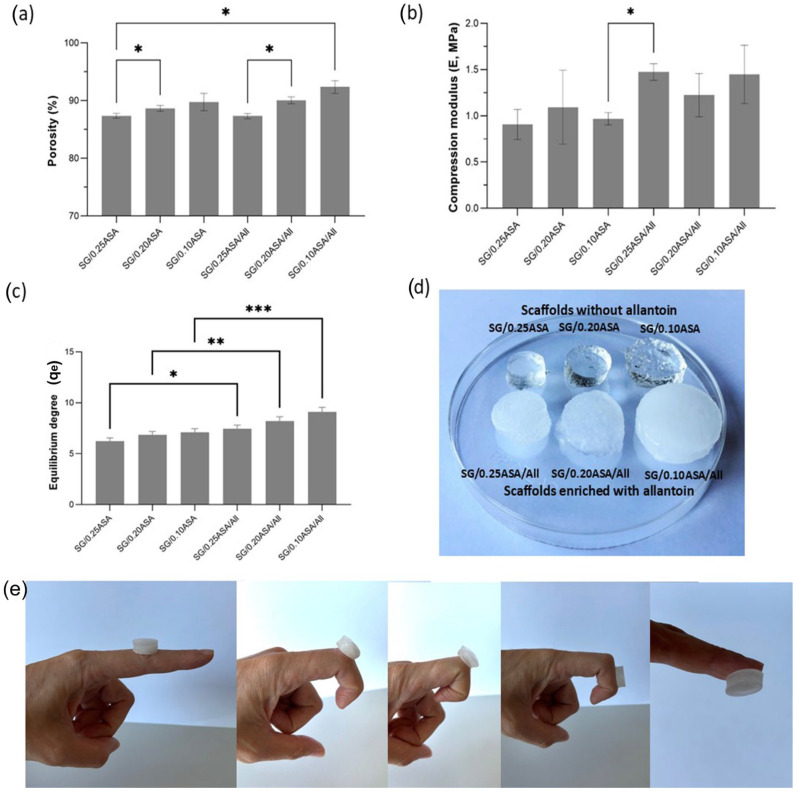
Obtained results of (**a**) porosity (%) (* *p* < 0.05), (**b**) compression modulus (*E*, MPa) (* *p* < 0.05), (**c**) equilibrium degree of swelling (*q_e_*) of the scaffolds (* *p* < 0.05, ** *p* < 0.01, and *** *p* < 0.001), (**d**) photographs of the swollen scaffolds, and (**e**) photographs of the scaffold attached to skin tissue at a moving joint, with bending angles varying from 0° to 120°, and at palm side of the finger.

Porosity is a key structural parameter that governs both the biological and mechanical performance of scaffolding biomaterials intended for tissue regeneration applications [48]. The obtained values of porosity of the gelatin/ascorbic acid-based scaffolds, as well as for those enriched with allantoin, are presented in Figure 3a. As can be seen, the porosity of the scaffolds is in the range from 87.37 ± 0.35 to 92.39 ± 0.89%, which is in accordance with the optimal interval reported for soft-tissue regeneration applications [49,50,51]. These results clearly demonstrate that scaffold composition significantly influences porosity. Thus, increasing the content of ASA in the scaffold composition resulted in reduced scaffold porosity, which can be attributed to a higher degree of crosslinking gelatin with a higher amount of ASA. As the degree of crosslinking increases, the polymer network becomes more compact and tightly organized [10]. Consequently, the available free volume within the structure decreases, which results in lower overall scaffold porosity In contrast, the scaffolds enriched with allantoin showed higher porosity compared with their counterparts without allantoin. This effect can be explained by the hydrophilic nature of allantoin, which enhances water uptake during scaffold preparation for lyophilization, leading to the formation of larger ice crystals during freeze drying and, consequently, producing scaffolds with larger pores [52]. Furthermore, the presence of allantoin may partially hinder the crosslinking process, resulting in a reduced degree of crosslinking of gelatin and, consequently, a more open porous structure. Overall, the scaffolds exhibited high values of porosity of approximately 90%, providing a favorable microenvironment for cell infiltration and tissue ingrowth while maintaining adequate structural integrity. These findings highlight the structural suitability of the gelatin/ascorbic acid scaffolds enriched with allantoin for soft-tissue regeneration applications and wound healing.

### 3.5. Mechanical Properties of the Scaffolds

The mechanical properties of scaffolds for tissue regeneration are a key parameter that must be precisely designed because they directly affect cellular functionality, tissue integration, and overall regenerative outcomes [8,53]. To effectively support skin tissue regeneration, the mechanical properties of scaffolds must be designed to replicate the native ECM of skin tissue, providing sufficient flexibility while maintaining structural integrity during handling and wound contraction [53]. Inadequate mechanical matching between a scaffold and native ECM of skin tissue—either excessive stiffness or insufficient strength—may compromise cellular responses, cause patient discomfort, or lead to scaffold failure [1,54]. Therefore, it is essential to characterize the mechanical properties of scaffolds for skin tissue regeneration and wound healing.

The obtained values of the compression modulus (*E*) of the gelatin/ascorbic acid scaffolds and their counterparts enriched with allantoin are presented in Figure 3b. The obtained values of the modulus *E* were in the range from 0.81 ± 0.18 to 1.47 ± 0.07 MPa, indicating dependence on the chemical composition of the scaffolds. The highest value of the elastic modulus (*E*) was observed for the SG/0.20ASA scaffold, suggesting that this gelatin-to-ASA ratio is optimal for enhancing the mechanical properties of gelatin scaffolds crosslinked with ascorbic acid. It was observed that the incorporation of allantoin significantly increased the values of *E*. This observation is in accordance with the obtained SEM micrographs for the scaffolds, which revealed thicker walls of pores in scaffolds enriched with allantoin. In addition, molecules of allantoin are presumed to occupy the free space in the polymeric network, thereby promoting a more compact and structurally cohesive matrix and, consequently, enhancing the mechanical strength of the scaffold. Consequently, allantoin acts as a reinforcing filler, enhancing the mechanical strength of the scaffolds compared with their allantoin-free counterparts. The obtained values of *E* indicate that the gelatin/ascorbic acid-based scaffolds, both with and without allantoin, exhibited soft-tissue-matched mechanical properties within a physiologically relevant range for skin tissue regeneration and wound healing, providing an appropriate balance between structural support and compliance required to mimic the native environment and to support cellular activities during the healing process [55].

### 3.6. Swelling Properties of the Scaffolds

The ability of scaffolds to absorb physiological fluids is an essential property for effective wound healing and skin tissue regeneration, as it regulates wound exudate and supports tissue repair. Insufficient fluid management can result in wound dehydration, while accumulation of excessive exudate can lead to tissue maceration and delayed healing [32]. The swelling behavior of the gelatin/ascorbic acid scaffolds, with and without allantoin, was assessed in vitro under simulated physiological pH and temperature conditions, and the obtained results, expressed as the values of the equilibrium degree of swelling (*q_e_)*, are summarized in Figure 3c. The *q_e_* values obtained for the scaffolds were in the range from 6.21 ± 0.31 to 9.12 ± 0.41, indicating that the increasing content of ascorbic acid decreased *q_e_* values, while the incorporation of allantoin increased the value of *q_e_*. This trend could be attributed to a higher degree of crosslinking of gelatin with a higher amount of ASA, which led to a reduced absorption capacity of the scaffolds, while the incorporation of allantoin, which is a hydrophilic compound, increased the absorption capacity of the scaffolds. The photographs of the scaffolds in the equilibrium state of swelling are presented in Figure 3d. The obtained *q_e_* values indicate that the absorption capacity of the scaffolds is primarily influenced by the degree of crosslinking, while the incorporation of a hydrophilic bioactive agent such as allantoin can enhance the swelling capacity of the scaffolds. This balance between crosslinking density and hydrophilicity allows the modulation of scaffold swelling capacity, which is important for effective exudate management in wound healing and skin tissue regeneration applications.

The relationship between the mechanical properties and swelling behavior of the polymeric scaffolds was strongly influenced by the degree of crosslinking and the hydrophilicity of the polymer network. Increasing the content of ascorbic acid (ASA) enhanced the degree of crosslinking within the gelatin-based network, which improved mechanical stiffness but reduced the free volume and mobility of polymeric chains, thereby limiting swelling capacity and resulting in lower *q_e_* values. In contrast, the incorporation of allantoin increased the hydrophilicity of the polymeric network due to its hydrophilic nature and promoted water absorption, while molecules of allantoin probably occupied free space within the polymeric network, contributing to a more compact, stable and mechanically stronger network. Consequently, allantoin contributed to both the enhanced swelling capacity and mechanical stability of the scaffold.

### 3.7. Skin Tissue Adhesion Properties of the Scaffolds

The tissue adhesive performance of scaffolds intended for skin tissue regeneration and wound healing is a crucial attribute, as it enables conformal attachment to irregular and dynamic wound surfaces, reduces the need for external fixation methods, and helps protect the wound from contamination and mechanical stress [56]. In this study, the adhesion of gelatin/ascorbic acid scaffolds, both with and without allantoin, was assessed by applying the swollen samples to skin tissue at a moving joint, with bending angles varying from 0° to 120° (Figure 3e). The scaffolds maintained stable attachment throughout the range of motion, demonstrating their potential for use on flexible skin regions. Scaffold adhesion is primarily driven by interactions at the interface between the scaffold surface and tissue, including hydrogen bonding, electrostatic interactions, and physical chain entanglement [57,58,59]. In the prepared scaffolds, gelatin provides numerous functional groups (e.g., amine, hydroxyl, and carboxyl groups) that can form hydrogen bonds with skin tissue components as well as electrostatic interactions with oppositely charged phospholipids in skin tissue membranes, thereby contributing to the stable adhesion of the scaffolds to the skin tissue [60,61]. Additionally, the incorporation of hydrophilic allantoin increases the swelling capacity of the scaffold, allowing it to absorb wound exudate/physiological fluids and form a thin hydration layer at the scaffold–tissue interface. This hydration layer promotes better interfacial contact, facilitating physical interactions, such as hydrogen bonding and van der Waals forces, between the scaffold surface and the tissue, which may enhance the overall adhesion of the scaffold to the tissue. The observed stable adhesion of the scaffolds to skin tissue under dynamic conditions confirms that the developed gelatin/ascorbic acid scaffolds enriched with allantoin possess suitable tissue-adhesive properties for wound healing and skin tissue regeneration applications without the need for additional fixation.

### 3.8. Biocompatibility Assays of the Scaffolds

**Figure 4:**
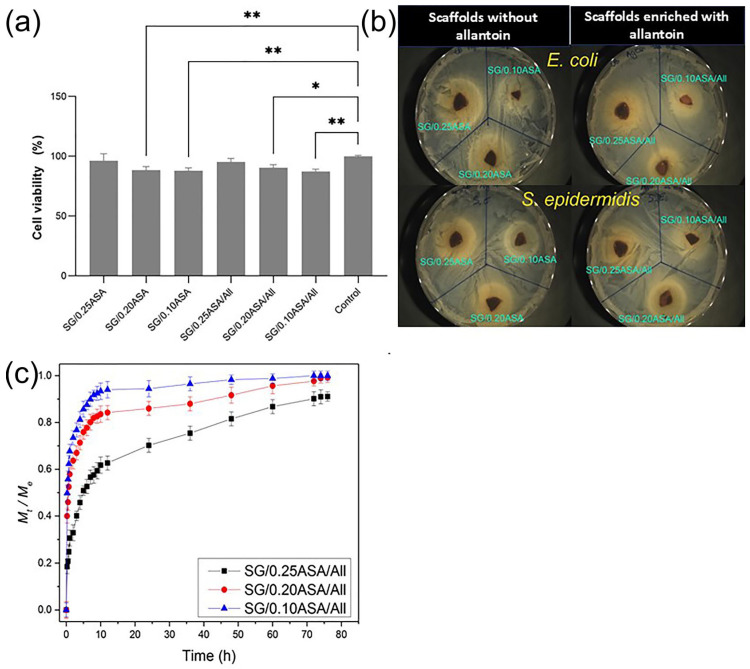
(**a**) HaCaT cell viability of the scaffolds (* *p* < 0.05 ** *p* < 0.01), (**b**) the antibacterial assay against *E. coli* and *S. epidermidis* bacteria (the inhibition zone around the scaffolds based on gelatin and ascorbic acid and those enriched with allantoin is evident in the presented images), and (**c**) the release profiles of allantoin obtained from scaffolds based on gelatin and ascorbic acid.

Cytocompatibility is a fundamental preliminary parameter that must be evaluated before a scaffold can be considered for in vivo application, including tissue engineering and wound healing [33,62]. The cytocompatibility of the developed gelatin/ascorbic acid scaffolds, and those enriched with allantoin, was evaluated using human keratinocyte (HaCaT) cells. HaCaT cells were selected due to keratinocytes representing the predominant cellular component of the epidermis and playing key roles in numerous physiological and pathological processes, including the regulation of complex mechanisms of wound healing, water and electrolyte balance, modulation of inflammatory and immune responses, and various mechanisms associated with carcinogenesis [63]. Additionally, HaCaT cells play a critical role in re-epithelialization and skin barrier restoration during wound healing and skin tissue regeneration [63]. The obtained results of the cytocompatibility of the scaffolds are presented in Figure 4a as percentages of cell viability. The results showed that all prepared scaffolds revealed a cell viability above 80% (ranging from 87.96 ± 1.87 to 96.31 ± 3.48%), confirming their biocompatibility and suitability for contact with skin cells. The observed moderate reduction in cell viability of the SG/0.20ASA and SG/0.10ASA scaffolds may reflect cellular adaptation to the scaffold environment or the influence of scaffold composition [64,65]. A clear influence of ASA content in the scaffold composition on the response of keratinocyte cells was observed, as the scaffold containing a higher ASA amount (SG/0.25ASA) showed a proliferative effect on HaCaT cells (up to 96.31%). Among the tested samples, SG/0.10ASA showed the lowest proliferative effect on HaCaT cells (87.96%). The incorporation of allantoin into the scaffolds did not significantly affect cellular responses, except for the SG/0.20ASA scaffold, where the incorporation of allantoin improved the proliferative effect compared with its allantoin-free counterpart. The observed favorable response of keratinocytes in contact with the scaffolds highlights the potential of the developed scaffolds as promising candidates for skin tissue regeneration and wound-healing applications.

### 3.9. Antibacterial Properties of the Scaffolds

Bacterial infection represents a major challenge in wound healing and skin tissue regeneration because it prolongs the inflammatory phase, disrupts collagen synthesis, impairs epidermal cell migration, and can lead to additional tissue damage and serious health consequences for patients [1,6]. For this reason, antibacterial activity is a key requirement in the design of wound dressings and scaffolds for skin tissue engineering. The microorganisms that are commonly associated with wound infection are *Staphylococcus epidermidis*, *Escherichia coli*, and *Pseudomonas aeruginosa* [6]. Accordingly, in the present study, the antibacterial activity of the prepared scaffolds was evaluated against both Gram-positive bacteria *S. epidermidis* and Gram-negative bacteria *E. coli* to assess their potential to control infection in wound healing and skin tissue regeneration applications.

The obtained results of the antibacterial assessment of the scaffolds are presented in Figure 4b. The inhibition zones around the scaffolds based on gelatin and ascorbic acid, and those enriched with allantoin, are evident in the presented images (Figure 4b), indicating that all synthesized scaffolds exhibited antibacterial activity against both tested bacteria. No bacterial growth was observed either directly underneath the scaffold samples or in their immediate environment, demonstrating both contact- and diffusion-based antimicrobial effects of the scaffolds. These findings indicate that the scaffolds based on gelatin and ascorbic acid and those enriched with allantoin are capable of suppressing bacterial growth and can, therefore, be considered as promising candidates for the treatment of infected wounds as well as for the prevention of secondary infections during wound healing and skin tissue regeneration.

### 3.10. In Vitro Allantoin Release Properties of the Scaffolds

The main concept of the proposed scaffold design is to provide both structural and bioactive support for wound healing and skin tissue regeneration within a single multifunctional system. In this approach, the highly porous 3D ECM-mimicking architecture serves as a physical framework that promotes cellular processes and tissue ingrowth, while the release of the incorporated bioactive agent provides biochemical stimulation of key regenerative mechanisms. Allantoin was the selected bioactive agent for incorporation into the 3D porous scaffold based on gelatin and ASA due to its known ability to stimulate proliferation of cells, modulate inflammatory responses, and enhance collagen and extracellular matrix synthesis [66,67]. To evaluate the bioactive functionality of the scaffolds enriched with allantoin, the in vitro allantoin release study was performed under simulated physiological conditions of pH and temperature, and the obtained release profiles are presented in Figure 4c. The obtained data demonstrated that scaffold composition strongly influenced release behavior. Scaffolds with higher ASA contents (SG/0.25ASA/All) exhibited a slower release, whereas those with lower ASA contents (SG/0.20ASA/All and SG/0.10ASA/All) displayed faster release kinetics. This phenomenon can be explained by differences in the swelling capacity of the scaffolds. The scaffolds obtained using a higher amount of ASA showed a lower equilibrium degree of swelling, probably due to the presence of crosslinks within the polymeric network, which hinder the mobility of polymeric chains and limit fluid uptake into the polymeric network. As a result, the dynamic of swelling and diffusion of active agents through the polymeric network occurs more gradually, leading to slower release of active agents. In contrast, scaffolds obtained with a lower content of ASA possess a higher equilibrium degree of swelling and a more porous, open, and flexible polymeric network architecture, which makes fluid uptake easier and promotes faster diffusion of active molecules through the polymeric network. This enhanced fluid transport contributes to faster release of active agents. During the initial phase of release (first 5 h), approximately 50% of allantoin was released from SG/0.25ASA/All, 75% from SG/0.20ASA/All, and 86% from SG/0.10ASA/All, highlighting the scaffold composition-dependent release pattern. This release trend can be attributed to differences in the degree of gelatin crosslinking induced by ASA content. Scaffolds with a higher degree of crosslinking exhibited reduced fluid uptake/limited swelling capacity, which hindered diffusion of allantoin through the polymeric network, resulting in slower release. In contrast, SG/0.10ASA/All, with a lower degree of crosslinking and higher swelling capacity, showed faster release of allantoin as the diffusion of allantoin through the swollen polymeric network is better. After the initial burst phase of release, all scaffolds exhibited a slower, sustained release of allantoin, while maintaining the same trend of release. Notably, the developed gelatin/ascorbic acid scaffolds enriched with allantoin provided release of allantoin for up to 76 h, indicating that the release profiles can be effectively tailored by adjusting scaffold composition. These tunable release kinetics, combined with the multifunctional properties of the scaffolds, highlight the potential of the proposed scaffolds as a promising strategy for improved skin tissue regeneration and wound healing.

## 4. Conclusions

This study demonstrates that fully natural gelatin/L-ascorbic acid scaffolds enriched with allantoin represent a promising multifunctional platform for advanced wound healing and skin tissue regeneration. The proposed simple preparation procedure by heat-induced crosslinking of gelatin with varying contents of ASA enabled the formation of a highly porous and interconnected ECM-replicated architecture with tunable porosity (87.37–92.39%) and compressive modulus values (0.81–1.47 MPa) within the range relevant for soft-tissue applications. Importantly, incorporation of allantoin improved scaffold performance by enhancing swelling capacity and mechanical properties. All prepared scaffolds exhibited antibacterial activity against both Gram-negative (*E. coli*) and Gram-positive (*S. epidermidis*) bacteria, indicating their ability to suppress bacterial colonization and support infection prevention during wound healing. Furthermore, the scaffolds exhibited good cytocompatibility toward human keratinocytes, with cell viability above 80% for all samples, thereby confirming their suitability for direct contact with skin tissue. The scaffolds showed sustained release of allantoin for up to 76 h, with tunable release kinetics by varying scaffold composition. These findings confirm that the developed scaffolds successfully integrate structural support, soft-tissue-matching mechanical properties, exudate management, antimicrobial functionality, keratinocyte-supportive properties, and controlled bioactive release within a single fully natural system, offering a biologically inspired strategy for improved skin regeneration and wound management.

## Figures and Tables

**Table 1:** The compositions and corresponding labels of the scaffolds based on gelatin and ascorbic acid and enriched with allantoin.

Scaffold	Gelatin (g)	L-Ascorbic Acid (g)	Allantoin (g)
SG/0.25ASA	2	0.5	0
SG/0.20ASA	2	0.4	0
SG/0.10ASA	2	0.2	0
SG/0.25ASA/All	2	0.5	1
SG/0.20ASA/All	2	0.4	1
SG/0.10ASA/All	2	0.2	1

## Data Availability

The original contributions presented in this study are included in the article. Further inquiries can be directed to the corresponding author.

## References

[1] Boateng J.S., Matthews K.H., Stevens H.N.E., Eccleston G.M. (2008). Wound healing dressings and drug delivery systems: A review. J. Pharm. Sci..

[2] Lazarus G.S., Cooper D.M., Knighton D.R., Margolis D.J., Pecoraro R.E., Rodeheaver G., Robson M.C. (1994). Definitions and guidelines for assessment of wounds and evaluation of healing. Arch. Dermatol.

[3] Da L.C., Huang Y.Z., Xie H.Q. (2017). Progress in development of bioderived materials for dermal wound healing. Regen. Biomater..

[4] Chaudhari A.A., Vig K., Baganizi D.R., Sahu R., Dixit S., Dennis V., Singh S.R., Pillai S.R. (2016). Future prospects for scaffolding methods and biomaterials in skin tissue engineering: A review. Int. J. Mol. Sci..

[5] Agarwal T., Narayan R., Maji S., Behera S., Kulanthaivel S., Maiti T.K., Banerjee I., Pal K., Giri S. (2016). Gelatin/carboxymethyl chitosan based scaffolds for dermal tissue engineering applications. Int. J. Biol. Macromol..

[6] Agrawal P., Soni S., Mittal G., Bhatnagar A. (2014). Role of polymeric biomaterials as wound healing agents. Int. J. Low. Extrem. Wounds.

[7] Huang S., Fu X. (2010). Naturally derived materials-based cell and drug delivery systems in skin regeneration. J. Control. Release.

[8] Sukmana I. (2012). Bioactive polymer scaffold for fabrication of vascularized engineering tissue. J. Artif. Organs.

[9] Demenj M., Žabčić M., Vukomanović M., Ilić-Tomić T., Milivojević D., Tomić S., Živanović D., Babić Radić M.M. (2025). Design of the multi-bioactive graphene-oxide/gelatin/alginate scaffolds as dual ECM-mimetic and specific wound healing phase-target therapeutic concept for advanced wound healing. Pharmaceutics.

[10] Babić Radić M.M., Žabčić M., Vukomanović M., Ilić-Tomić T., Milivojević D., Tomić S., Živanović D., Radić M.M.B. (2025). Development of nano ZnO-embedded gelatin/alginate bioscaffolds for potential skin tissue regeneration via oxidative stress modulation and ECM mimicry. Biopolymers.

[11] Poursamar S.A., Hatami J., Lehner A.N., Da Silva C.L., Ferreira F.C., Antunes A.P.M. (2016). Potential application of gelatin scaffolds prepared through in situ gas foaming in skin tissue engineering. Int. J. Polym. Mater. Polym. Biomater..

[12] Yang G., Xiao Z., Long H., Ma K., Zhang J., Ren X., Zhang J. (2018). Assessment of the characteristics and biocompatibility of gelatin sponge scaffolds prepared by various crosslinking methods. Sci. Rep..

[13] Shankar K.G., Gostynska N., Montesi M., Panseri S., Sprio S., Kon E., Marcacci M., Tampieri A., Sandri M. (2017). Investigation of different cross-linking approaches on 3D gelatin scaffolds for tissue engineering application: A comparative analysis. Int. J. Biol. Macromol..

[14] Kumar P.T.S., Praveen G., Raj M., Chennazhi K.P., Jayakumar R. (2014). Flexible, micro-porous chitosan-gelatin hydrogel/nanofibrin composite bandages for treating burn wounds. RSC Adv..

[15] Ghalei S., Nourmohammadi J., Solouk A., Mirzadeh H. (2018). Enhanced cellular response elicited by addition of amniotic fluid to alginate hydrogel–electrospun silk fibroin fibers for potential wound dressing application. Colloids Surf. B.

[16] Babić Radić M.M., Vukomanović M., Nikodinović-Runić J., Tomić S. (2024). Gelatin-/Alginate-Based Hydrogel Scaffolds Reinforced with TiO2 Nanoparticles for Simultaneous Release of Allantoin, Caffeic Acid, and Quercetin as Multi-Target Wound Therapy Platform. Pharmaceutics.

[17] Babić Radić M.M., Filipović V.V., Vukomanović M., Nikodinović-Runić J., Tomić S.L. (2021). Degradable 2-Hydroxyethyl Methacrylate/Gelatin/Alginate Hydrogels Infused by Nanocolloidal Graphene Oxide as Promising Drug Delivery and Scaffolding Biomaterials. Gels.

[18] Takigawa T., Endo Y. (2006). Effects of glutaraldehyde exposure on human health. J. Occup. Health.

[19] Li J., Ren N., Qiu J., Jiang H., Zhao H., Wang G., Boughton R.I., Wang Y., Liu H. (2013). Carbodiimide crosslinked collagen from porcine dermal matrix for high-strength tissue engineering scaffold. Int. J. Biol. Macromol..

[20] Zeiger E., Gollapudi B., Spencer P. (2005). Genetic toxicity and carcinogenicity studies of glutaraldehyde—A review. Mutat. Res..

[21] Pischetsrieder M. (1996). Reaction of L-ascorbic acid with L-arginine derivatives. J. Agric. Food Chem..

[22] Linda M. (1991). Nutritional Biochemistry and Metabolism with Clinical Applications.

[23] Delafuente J.C., Prendergast J.M., Modigh A. (1986). Immunologic modulation by vitamin C. Int. J. Immunopharmacol..

[24] Larisch B., Gross U., Pischetsrieder M. (1998). On the reaction of L-ascorbic acid with propylamine under various conditions: Quantification of the main products by HPLC/DAD. Z. Lebensm. Unters. Forsch. A-Food Res. Technol..

[25] Tiller J., Berlin P., Klemm D. (1999). A novel efficient enzyme-immobilization reaction on NH_2_ polymers by means of L-ascorbic acid. Biotechnol. Appl. Biochem..

[26] Falconi M., Salvatore V., Teti G., Focaroli S., Durante S., Nicolini B., Mazzotti A., Orienti I. (2013). Gelatin crosslinked with dehydroascorbic acid as a novel scaffold for tissue regeneration with simultaneous antitumor activity. Biomed. Mater..

[27] Wilson J.X. (2002). The physiological role of dehydroascorbic acid. FEBS Lett..

[28] Cárcamo J.M., Pedraza A., Bórquez-Ojeda O., Zhang B., Sanchez R., Golde D.W. (2004). Vitamin C is a kinase inhibitor: Dehydroascorbic acid inhibits IκB-alpha kinase beta. Mol. Cell. Biol..

[29] Vera J.C., Rivas C.I., Fischbarg J., Golde D.W. (1993). Mammalian facilitative hexose transporters mediate the transport of dehydroascorbic acid. Nature.

[30] Robinson W. (1935). Stimulation of healing wounds: By allantoin occurring in maggot secretions and of wide biological distribution. J. Bone Jt. Surg..

[31] Araújo L.U., Grabe-Guimarães A., Mosqueira V.C.F., Carneiro C.M., Silva-Barcellos N.M. (2010). Profile of wound healing process induced by allantoin. Acta Cir. Bras..

[32] Yaşayan G., Karaca G., Akgüner Z.P., Bal Öztürk A. (2020). Chitosan/collagen composite films as wound dressings encapsulating allantoin and lidocaine hydrochloride. Int. J. Polym. Mater. Polym. Biomater..

[33] Sakthiguru N., Sithique M.A. (2020). Fabrication of bioinspired chitosan/gelatin/allantoin biocomposite film for wound dressing application. Int. J. Biol. Macromol..

[34] Babić M.M., Vukomanović M., Stefanič M., Nikodinović-Runić J., Tomić S.L. (2020). Controlled curcumin release from hydrogel scaffold platform based on 2-hydroxyethyl methacrylate/gelatin/alginate/iron(III) oxide. Macromol. Chem. Phys..

[35] Bell C.L., Peppas N.A. (1995). Measurement of swelling force in ionic polymer networks. III. Swelling force of interpolymer complexes. J. Control. Release.

[36] Peppas N.A. (1985). Analysis of Fickian and non-Fickian drug release from polymers. Pharm. Acta Helv..

[37] Babić M.M., Antić K.M., Vuković J.S., Božić B.Đ., Davidović S.Z., Filipović J.M., Tomić S.L. (2015). Oxaprozin/poly(2-hydroxyethyl acrylate/itaconic acid) hydrogels: Morphological, thermal, swelling, drug release and antibacterial properties. J. Mater. Sci..

[38] Fei F., Sanjoy S., Donny H.P. (2021). Biomimetic hydrogels to promote wound healing. Front. Bioeng. Biotechnol..

[39] Koosha M., Aalipour H., Sarraf Shirazi M.J., Jebali A., Chi H., Hamedi S., Wang N., Li T., Moravvej H. (2021). Physically crosslinked chitosan/PVA hydrogels containing honey and allantoin with long-term biocompatibility for skin wound repair: An in vitro and in vivo study. J. Funct. Biomater..

[40] Pischetsrieder M., Larisch B., Severin T., O’Brien J., Nursten H.E., Crabbe M.J.C., Ames J.M. (2005). The Maillard Reaction of Ascorbic Acid with Amino Acids and Proteins—Identification of Products. The Maillard Reaction in Foods and Medicine.

[41] Cheah Y.J., Yunus M.H.M., Fauzi M.B., Tabata Y., Hiraoka Y., Phang S.J., Chia M.R., Buyong M.R., Yazid M.D. (2023). Gelatin–chitosan–cellulose nanocrystals as an acellular scaffold for wound healing application: Fabrication, characterisation and cytocompatibility towards primary human skin cells. Cellulose.

[42] Tian X., Tian D., Wang Z.-Y., Mo F. (2009). Synthesis and evaluation of chitosan-vitamin C complex. Indian J. Pharm. Sci..

[43] Zhong S., Li B., Ji Y., Zeng C. (2016). Multifunctional coordination polymer nanoparticles based on allantoin: Single peak upconversion emission, drug delivery and cytotoxicity study. J. Inorg. Organomet. Polym..

[44] Menezes J.E.S.A., dos Santos H.S., Ferreira M.K.A., Magalhães F.E.A., da Silva D.S., Bandeira P.N., Saraiva G.D., Pessoa O.D.L., Ricardo N.M.P.S., Cruz B.G. (2020). Preparation, structural and spectroscopic characterization of chitosan membranes containing allantoin. J. Mol. Struct..

[45] Annabi N., Nichol J.W., Zhong X., Ji C., Koshy S., Khademhosseini A., Dehghani F. (2010). Controlling the porosity and microarchitecture of hydrogels for tissue engineering. Tissue Eng. B Rev..

[46] Shi C., Yuan Z., Han F., Zhu C., Li B. (2016). Polymeric biomaterials for bone regeneration. Ann. Jt..

[47] Raucci M.G., D’Amora U., Ronca A., Demitri C., Ambrosio L. (2019). Bioactivation routes of gelatin-based scaffolds to enhance at nanoscale level bone tissue regeneration. Front. Bioeng. Biotechnol..

[48] Carvalho I.C. (2017). Engineered 3D scaffolds of photocrosslinked chitosan-gelatin hydrogel hybrids for chronic wound dressings and regeneration. Mater. Sci. Eng. C.

[49] Vadav P., Beniwal G., Saxena K.K. (2021). A review on pore and porosity in tissue engineering. Mater. Today Proc..

[50] Staruch R.M., Glass G.E., Rickard R., Hettiaratchy S.P., Butler P.E.M. (2017). Injectable pore-forming hydrogel scaffolds for complex wound tissue engineering: Designing and controlling their porosity and mechanical properties. Tissue Eng. Part B Rev..

[51] Zang J.C., Wu L.B., Jing D.Y., Ding J.D. (2005). A comparative study of porous scaffolds with cubic and spherical macropores. Polymer.

[52] Azizian S., Hadjizadeh A., Niknejad H. (2018). Chitosan-gelatin porous scaffold incorporated with chitosan nanoparticles for growth factor delivery in tissue engineering. Carbohydr. Polym..

[53] Cai N., Li C., Han C., Luo X., Shen L., Xue Y., Yu F. (2016). Tailoring mechanical and antibacterial properties of chitosan/gelatin nanofiber membranes with Fe_3_O_4_ nanoparticles for potential wound dressing application. Appl. Surf. Sci..

[54] Tseng H.J., Tsou T.L., Wang H.J., Hsu S.H. (2013). Characterization of chitosan-gelatin scaffolds for dermal tissue engineering. J. Tissue Eng. Regen. Med..

[55] Carton F., Rizzi M., Canciani E., Sieve G., Di Francesco D., Casarella S., Di Nunno L., Boccafoschi F. (2024). Use of hydrogels in regenerative medicine: Focus on mechanical properties. Int. J. Mol. Sci..

[56] Hou Y., Jiang N., Sun D., Wang Y., Chen X., Zhu S., Zhang L. (2020). A fast UV-curable PU-PAAm hydrogel with mechanical flexibility and self-adhesion for wound healing. RSC Adv..

[57] Nishiguchi A., Kurihara Y., Taguchi T. (2019). Underwater-adhesive microparticle dressing composed of hydrophobically-modified Alaska pollock gelatin for gastrointestinal tract wound healing. Acta Biomater..

[58] Simson J.A., Strehin I.A., Allen B.W., Elisseeff J.H. (2013). Bonding and fusion of meniscus fibrocartilage using a novel chondroitin sulfate bone marrow tissue adhesive. Tissue Eng. Part A.

[59] Korde M.J., Kandasubramanian B. (2018). Biocompatible alkyl cyanoacrylates and their derivatives as bio-adhesives. Biomater. Sci..

[60] Qu J., Zhao X., Liang Y., Zhang T., Ma P.X., Guo B. (2018). Antibacterial adhesive injectable hydrogels with rapid self-healing, extensibility and compressibility as wound dressing for joint skin wound healing. Biomaterials.

[61] Chen S., Gil C.J., Ning L., Jin L., Perez L., Kabboul G., Tomov M.L., Serpooshan V. (2021). Adhesive tissue engineered scaffolds: Mechanisms and applications. Front. Bioeng. Biotechnol..

[62] Yang C., Xu L., Zhou Y., Zhang X., Huang X., Wang M., Han Y., Zhai M., Wei S., Li J. (2010). A green fabrication approach of gelatin/CM-chitosan hybrid hydrogel for wound healing. Carbohydr. Polym..

[63] Al-Dhubaibi M.S., Mohammed G.F., Bahaj S.S., AbdElneam A.I., Al-Dhubaibi A.M., Atef L.M. (2025). The role of keratinocytes in skin health and disease. Dermatol. Rev..

[64] Li W., Zhou J., Xu Y. (2015). Study of the in vitro cytotoxicity testing of medical devices. Biomed. Rep..

[65] (2009). Biological evaluation of medical devices—Part 5: Tests for in vitro cytotoxicity.

[66] Alam R., Rasheed R., Ashraf M.A., Hussain I., Ali S. (2023). Allantoin alleviates chromium phytotoxic effects on wheat by regulating osmolyte accumulation, secondary metabolism, ROS homeostasis and nutrient acquisition. J. Hazard. Mater..

[67] Tzeng C.Y., Lee W.S., Liu K.F., Tsou H.K., Chen C.J., Peng W.H., Tsai J.C. (2022). Allantoin ameliorates amyloid β-peptide-induced memory impairment by regulating the PI3K/Akt/GSK-3β signaling pathway in rats. Biomed. Pharmacother..

